# Prediction of soil cadmium distribution across a typical area of Chengdu Plain, China

**DOI:** 10.1038/s41598-017-07690-y

**Published:** 2017-07-28

**Authors:** Qiquan Li, Changquan Wang, Tianfei Dai, Wenjiao Shi, Xin Zhang, Yi Xiao, Weiping Song, Bing Li, Yongdong Wang

**Affiliations:** 1 College of Resources, Sichuan Agricultural University, Chengdu, 611130 China; 2 Chengdu Testing Center of Soil and Fertilizer, Chengdu, 610041 China; 3 Key Laboratory of Land Surface Pattern and Simulation, Institute of Geographic Sciences and Natural Resources Research, Chinese Academy of Sciences, Beijing, 100101 China; 4 College of Resources and Environment, University of Chinese Academy of Sciences, Beijing, 100049 China; 5 Department of Transportation of Sichuan Province, Chengdu, 610041 China

## Abstract

A suitable method and appropriate environmental variables are important for accurately predicting heavy metal distribution in soils. However, the classical methods (e.g., ordinary kriging (OK)) have a smoothing effect that results in a tendency to neglect local variability, and the commonly used environmental variables (e.g., terrain factors) are ineffective for improving predictions across plains. Here, variables were derived from the obvious factors affecting soil cadmium (Cd), such as road traffic, and were used as auxiliary variables for a combined method (HASM_RBFNN) that was developed using high accuracy surface modelling (HASM) and radial basis function neural network (RBFNN) model. This combined method was then used to predict soil Cd distribution in a typical area of Chengdu Plain in China, considering the spatial non-stationarity of the relationships between soil Cd and the derived variables based on 339 surface soil samples. The results showed that HASM_RBFNN had lower prediction errors than OK, regression kriging (RK) and HASM_RBFNN_s_, which didn’t consider the spatial non-stationarity of the soil Cd-derived variables relationships. Furthermore, HASM_RBFNN provided improved detail on local variations. The better performance suggested that the derived environmental variables were effective and HASM_RBFNN was appropriate for improving the prediction of soil Cd distribution across plains.

## Introduction

Heavy metals in the soil are crucial factors of environmental and food quality and can threaten human health through the food chain^1, 2^. In recent decades, heavy metal pollution of soils has become a globally recognized environmental issue^1, 3, 4^. To evaluate the potential risks to humans and the environment, there is a growing concern about the spatial distributions of soil heavy metals in the environment because an inaccurate estimation of soil heavy metal distributions will result in considerable bias in risk assessment^1^. Soil sampling analysis can provide highly accurate data of soil heavy metals at sampling sites, but these sampling points are sparse because of the laborious sampling process and the expensive costs for sample analysis in the lab^5^. Therefore, methods of spatial distribution modelling are required to obtain accurate spatial distribution maps of soil heavy metals from limited point observations for risk control.

Several classical methods, such as kriging, inverse distance weighting and splines, are extensively used to estimate the spatial distributions of soil heavy metals in soil pollution investigations^1^; nevertheless, each of these methods has its own limitations^1, 5^. These classical methods, which predict the soil heavy metal contents of untested locations based on the neighbouring soil samples and the spatial autocorrelation of soil sampling data^6^, all have a smoothing effect that tends to underestimate the local high values and overestimate the local low values^7^. This smoothing effect may result in a failure to recognize local variation and thereby produce inaccurate spatial distributions of soil heavy metals in the soil pollution assessment process, which can affect relevant environmental decisions^1^. As a result of higher population densities, more intensive agricultural practices, rapid urbanization and industrialization, as well as natural sources, heavy metal pollution has become quite serious and the spatial distributions of heavy metals tend to be more complex across plains^1, 8–11^. Previous studies of soil heavy metals across plains have shown that anthropogenic factors, such as roads and crop rotation systems, as well as geogenic sources, can have substantial impacts on soil heavy metal content and can lead to high local variability in soil heavy metal distributions^9, 11^. Due to the limitations of the classical methods, a more effective spatial distribution modelling method is needed for predicting soil heavy metal distributions across plains.

In recent studies, a methodological framework that predicts the spatial distribution of soil properties based on both the environmental correlation between a soil variable and environmental parameters and the spatial autocorrelation in the residuals of the soil variable has been proven to be effective for obtaining more accurate spatial information on soil properties and has received increasing attention^5, 6, 12–18^. With this framework, a combined method is developed based on the premise that the deterministic component of the targeted soil variable caused by correlated environmental factors can be explained by a regression model while the spatially varying but dependent component can be described by the prediction residuals of the linear regression model and captured by the classical methods such as ordinary kriging (OK)^6, 13, 15–19^. For instance, regression kriging (RK), which has been widely employed in many studies^6, 15, 16, 19^, is the typical combined method that uses multiple linear regression model (MLR) to capture the relationships between soil and the environmental factors and uses OK to interpolate regression residuals to prediction grids.

However, the commonly used factors, such as terrain factors, land use and soil type, cannot effectively reproduce the spatial variability of the soil heavy metals in plains because of the gently undulating terrain, the relatively homogeneous parent material and soil type, and other anthropogenic factors such as traffic road^9, 11^ and different rotation systems of farmland^11, 20, 21^ that have great impacts on soil heavy metals. Therefore, new environmental covariates rather than the above commonly used environmental factors should be employed as auxiliary variables in predicting soil heavy metal distributions across plains. Moreover, the relationships between soils and environmental factors are often nonlinear and spatial non-stationary, suggesting that the nonlinear relationships vary across space^6, 12^; thus, a single linear regression model is unlikely to effectively capture such complex relationships for all subareas in a regional study^14^. In addition, although OK is the most commonly used classical method in soil science and can provide the best linear unbiased estimates, this method is based on an assumption (intrinsic stationarity) that may not be met in practice^22^. Recent studies have found that the radial basis function neural network (RBFNN) approach can perform better than MLR due to its capacity to capture the complex relationships between soils and the environmental factors^12, 15^, and a new approach, called high accuracy surface modelling (HASM), developed on the basis of a fundamental theorem of surfaces by Yue *et al*.^23–26^, can outperform the three classical methods for predicting soil properties^16, 22^. Both approaches provide new tools for predicting soil heavy metal distributions across plains based on the methodological framework of the combined methods.

Cadmium (Cd) is an extremely important pollutant among the various heavy metal elements because of its high transfer rate from soil to plants and strong bio-toxicity^27, 28^. Soil Cd has become an important environmental pollutant around the world^2, 4, 28–30^ and was also found to be a serious pollutant on the Chengdu Plain of China^11, 31, 32^. Previous studies have shown that geological origin, road distribution and crop rotation systems had great influences on soil Cd in the farmland of the Chengdu Plain^11, 32^. This study aimed to develop a method to predict soil Cd distribution in a central area of the Chengdu Plain. The specific objectives were (1) to derive new environmental variables from the factors noted above; (2) to develop a combined method (HASM_RBFNN) using HASM and RBFNN to predict the spatial distribution of soil Cd that considers the nonlinearity and the spatial non-stationarity of the relationships between soil Cd and the derived environmental covariates; and (3) to evaluate its performance compared with that of the OK, RK and HASM_RBFNN_s_ which did not consider the spatial non-stationarity of the relationships.

## Results

### Correlation between soil Cd and the environmental covariates

**Figure 1:**
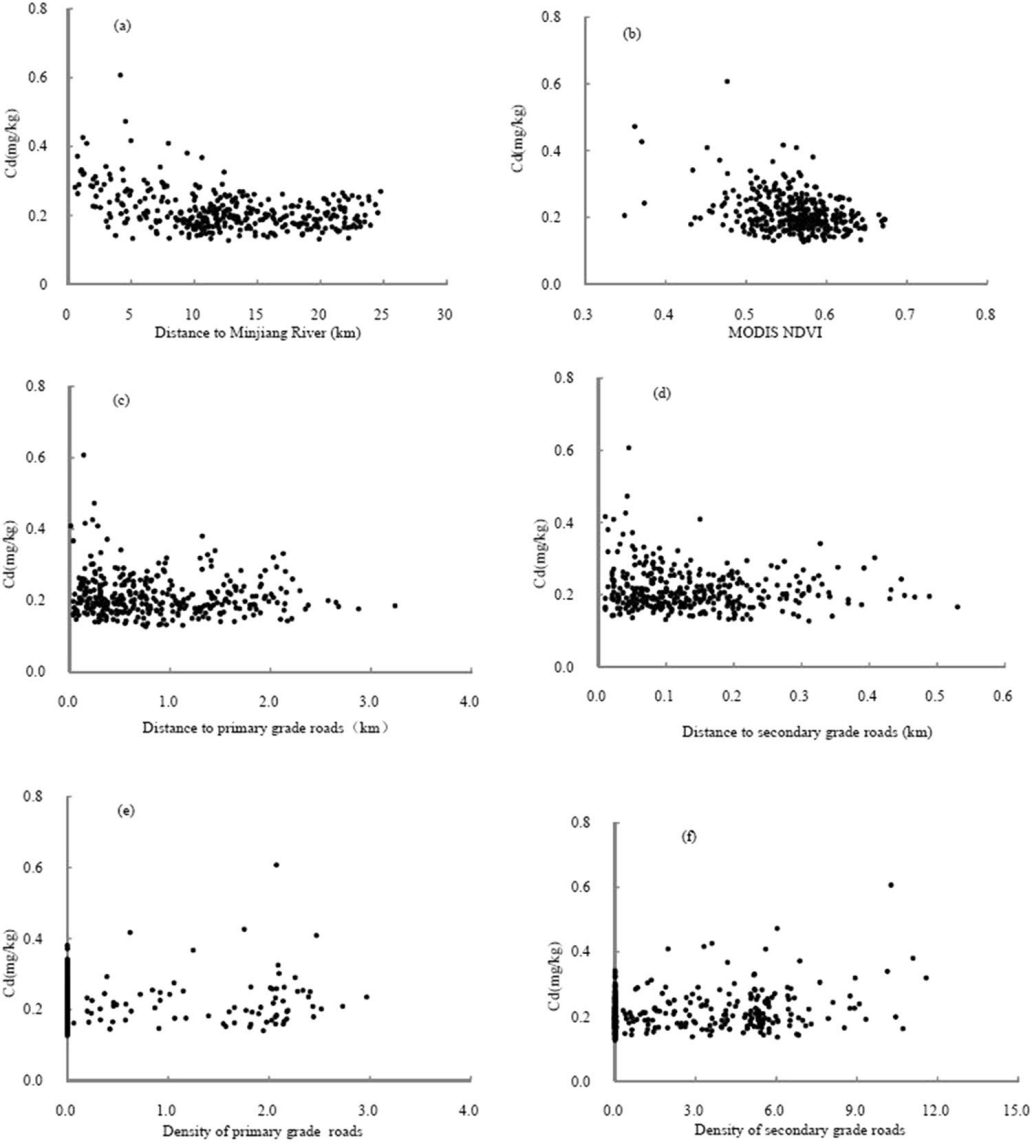
Relationships between soil Cd content and distance to the Minjiang River (**a**), MODIS NDVI (**b**), the distances to primary (**c**) and secondary (**d**) grade roads, and the density of primary (**e**) and secondary (**f**) grade roads.

The relationships between soil Cd and the environmental factors are shown in Fig. 1a–f. Soil Cd content was negatively correlated with the distance to the Minjiang River; soil Cd content declined significantly with increasing distance to the Minjiang River within 10 km of the Minjiang River (Fig. 1a). Moderate-Resolution Imaging Spectroradiometer (MODIS) normalized difference vegetation index (NDVI) also showed a negative correlation with soil Cd (Fig. 1b). Primary and secondary roads had impacts on soil Cd up to approximately 1.2 km and 0.2 km from the roads, respectively, and the impacts were more significant within 0.5 km and 0.1 km of these types of roads (Fig. 1c and d), which led to positive correlations between soil Cd and the densities of the two grades of roads within a 500 × 500 m and a 100 × 100 m grid, respectively (Fig. 1e and f).

According to regression analysis (Table 1), the four derived factors all had significant impacts on soil Cd (*p* < 0.05 or *p* < 0.01). Distance to the Minjiang River, MODIS NDVI and the densities of primary and secondary roads contributed 22.0%, 12.8%, 1.4% and 5.7% of soil Cd variability, respectively. However, the correlations changed in different subareas. Within 10 km of the Minjiang River, soil Cd showed significant negative correlations with MODIS NDVI and the distance to the Minjiang River and positive correlations with the densities of both grades of roads, while soil Cd only showed significant correlations with MODIS NDVI and the density of primary roads beyond 10 km of the Minjiang River (Table 2).

**Table 1:** Results of regression analysis using different factors as independent variables.

Factors	Regression equation	*R* ^2^	*F*	*p*
Distance to the Minjiang River	Y = −0.039 ln (X) + 0.306	0.220	94.908	<0.01
MODIS NDVI	Y = −0.423X + 0.451	0.128	49.354	<0.01
Density of primary roads	Y = 0.009X + 0.211	0.014	4.931	0.027
Density of secondary roads	Y = 0.005X + 0.202	0.057	20.277	<0.01

**Table 2:** Relationships between soil Cd content and the factors in different subareas.

Subareas	Sample number	Distance to the Minjiang River	MODIS NDVI	Density of primary roads	Density of secondary roads
Within 10 km of the Minjiang River	84	−0.44^**^	−0.64^**^	0.25^*^	0.43^**^
Beyond 10 km of the Minjiang River	189	−0.02	−0.12^*^	0.14^**^	−0.01

### Comparison of the prediction accuracies of different methods

The prediction errors, including the mean absolute error (MAE), root mean square error (RMSE) and mean relative error (MRE), of different methods for the independent validation points are listed in Table 3. The results indicated that HASM_RBFNN could achieve the smallest prediction errors, followed by HASM_RBFNNs, RK and OK, indicating that HASM_RBFNN was the most accurate method and the derived factors and the selected approached for establishing the combined method could contribute to the improved prediction accuracy of soil Cd distribution across the study area.

**Table 3:** Prediction errors of the different methods for the independent validation points. MAE, mean absolute error; RMSE, root mean square error; MRE, mean relative error; OK, ordinary kriging; RK, regression kriging; HASM_RBFNN, the combined method (HASM_RBFNN) developed using high-accuracy surface modelling (HASM) and radial basis function neural network (RBFNN) modelling, taking into account the spatial non-stationarity of the relationships between soil Cd and the auxiliary variables; HASM_ RBFNNs, the combined method (HASM_RBFNN), without taking into account the spatial non-stationarity of the relationships.

Methods	Sample number	MAE	RMSE	MRE
HASM_RBFNN	66	0.034	0.042	15.715
HASM_RBFNNs	66	0.036	0.045	16.622
RK	66	0.037	0.046	17.746
OK	66	0.040	0.051	18.083

### Comparison of the prediction maps created by different methods

**Figure 2:**
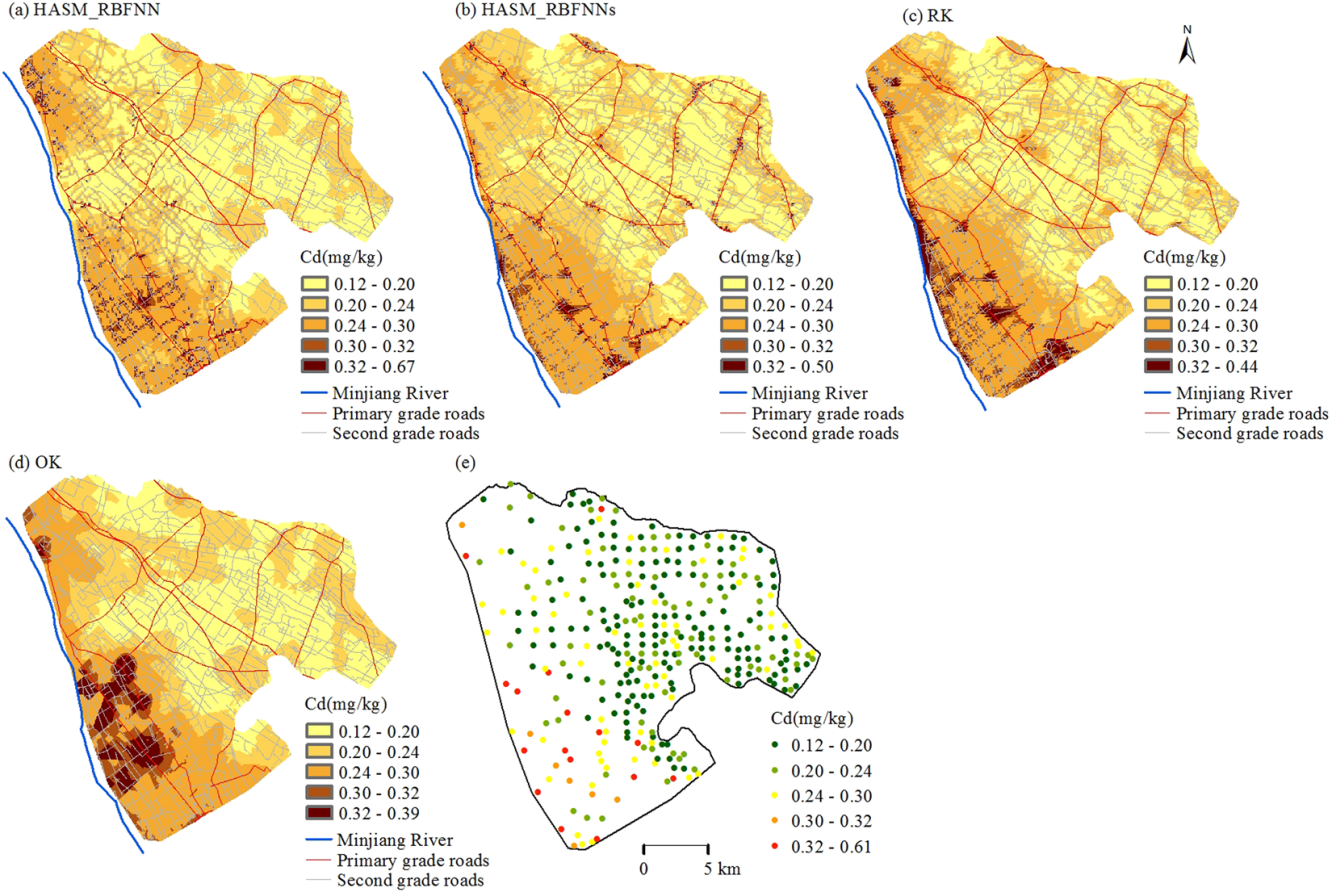
The spatial distribution maps by HASM_RBFNN (**a**), HASM_RBFNNs (**b**), RK (**c**) and OK (**d**). (OK, ordinary kriging; RK, regression kriging; HASM_RBFNN, the combined method (HASM_RBFNN) developed using high-accuracy surface modelling (HASM) and radial basis function neural network (RBFNN) modelling, taking into account the spatial non-stationarity of the relationships between soil Cd and the auxiliary variables; HASM_RBFNNs, the combined method (HASM_RBFNN), without taking into account the spatial non-stationarity of the relationships.). All the maps were generated in ArcGIS10.1, http://www.esrichina-bj.cn/softwareproduct/ArcGIS/.

The spatial distribution maps of soil Cd predicted by the four methods are illustrated in Fig. 2 (a–d). The prediction maps of soil Cd distribution obtained from the four methods exhibited similar spatial patterns, which showed that soil Cd was relatively higher in the subarea that is closest to the Minjiang River. However, differences among the prediction results of the four methods were obvious (Fig. 2). HASM_RBFNN obtained the largest prediction ranges that were closest to the observed values, followed by HASM_RBFNNs and RK, while OK had the narrowest prediction ranges among the methods. The prediction map produced by OK showed rather gradual transitions with limited detail and could not accurately reproduce the local variability (Fig. 2d). Conversely, HASM_RBFNN, HASM_RBFNNs and RK performed better, with more detail in the prediction results (Fig. 2a–c), indicating that the methods utilizing the derived environmental covariates as auxiliary variables could significantly improve the performance of local variability reproduction. Moreover, OK produced soil Cd maps with much larger areas of high value (>0.32 mg·kg^−1^), indicating that the points with high values of soil Cd had great impacts on the prediction results surrounding these points, which overestimated the soil Cd contents of these areas. Prediction maps by HASM_RBFNNs and RK showed some areas with soil Cd exceeding 0.32 mg·kg^−1^ along roads in the eastern region of the study area, which was inconsistent with the measured data (Fig. 2e), while the prediction map by HASM_RBFNN was more consistent with the actual soil Cd distribution (Fig. 2a).

## Discussion

### Effects of environmental factors on soil Cd

According to the semivariogram analysis (Table 4), the ratio of nugget to sill was 0.437, suggesting that soil Cd in this study area was determined by the combined effects of natural and anthropogenic sources. Regression analysis (Table 1) further indicated that a natural factor (distance to the Minjiang River) was a more important factor than the anthropogenic sources including roads and MODIS NDVI that could reflect the differences between rice-wheat and rice-rapeseed rotation systems based on our calculation method. This result was in agreement with previous studies on the Chengdu Plain^11, 32, 33^.

**Table 4:** Semivariogram parameters of soil Cd and the regression residuals.

Variables	Models	Nugget (C_0_)	Sill (C_0_ + C)	Nugget/sillC_0_/(C_0_ + C)	Range (km)	R^2^
Soil Cd	Exponential	0.038	0.087	0.437	25.2	0.968
Regression residuals	Gaussian	0.0075	0.017	0.441	20.7	0.913

The soil parent material of the study area mainly arrives via river transportation from the Longmen Mountains, located to the northwest, where the geological background value of soil Cd is 0.376 mg.kg^−1^; in fact, the Cd contents of Carboniferous, Devonian and Sinian outcrops in this mountain range can be up to 0.659 mg.kg^−1^ ^11, 34^. A previous study also showed that Cd contents in the first terrace and stream sediment of the Minjiang River were 0.27 and 0.53 mg.kg^−1^, respectively^35^. The distance from the Minjiang River reflects the differences of the sedimentation process of the parent material and the formation time of the soil. The shorter the distance, the more recent the soil deposition and the more similar the soil to the parent material, which may account for the fact that soil Cd content negatively correlated with the distance to the Minjiang River (Fig. 1a).

The differences between the two rotation systems were related to the different management measures pertaining to fertilizers, pesticides and the aboveground straws. For example, wheat straw was often returned to the field, while rapeseed straw was always removed in this area. Furthermore, previous studies have indicated that the Cd content in rapeseed is larger than in wheat^21, 22^. The different management measures for aboveground straw, different Cd content and the different biomass of wheat and rapeseed may partially result in higher soil Cd content in the rice-wheat rotation systems regardless of any differences in fertilizer management^11^. In this study, the average MODIS NDVI, calculated from the MODIS NDVI of February, July and December of 2006 to 2012, could reflect the differences in the vegetation cover between the two rotation systems as well as soil conditions, where high NDVI values correspond to rice-rapeseed rotation systems and low NDVI values may correspond to rice-wheat rotation systems. This condition led to the negative correlation between soil Cd and MODIS NDVI (Fig. 1b).

**Figure 3:**
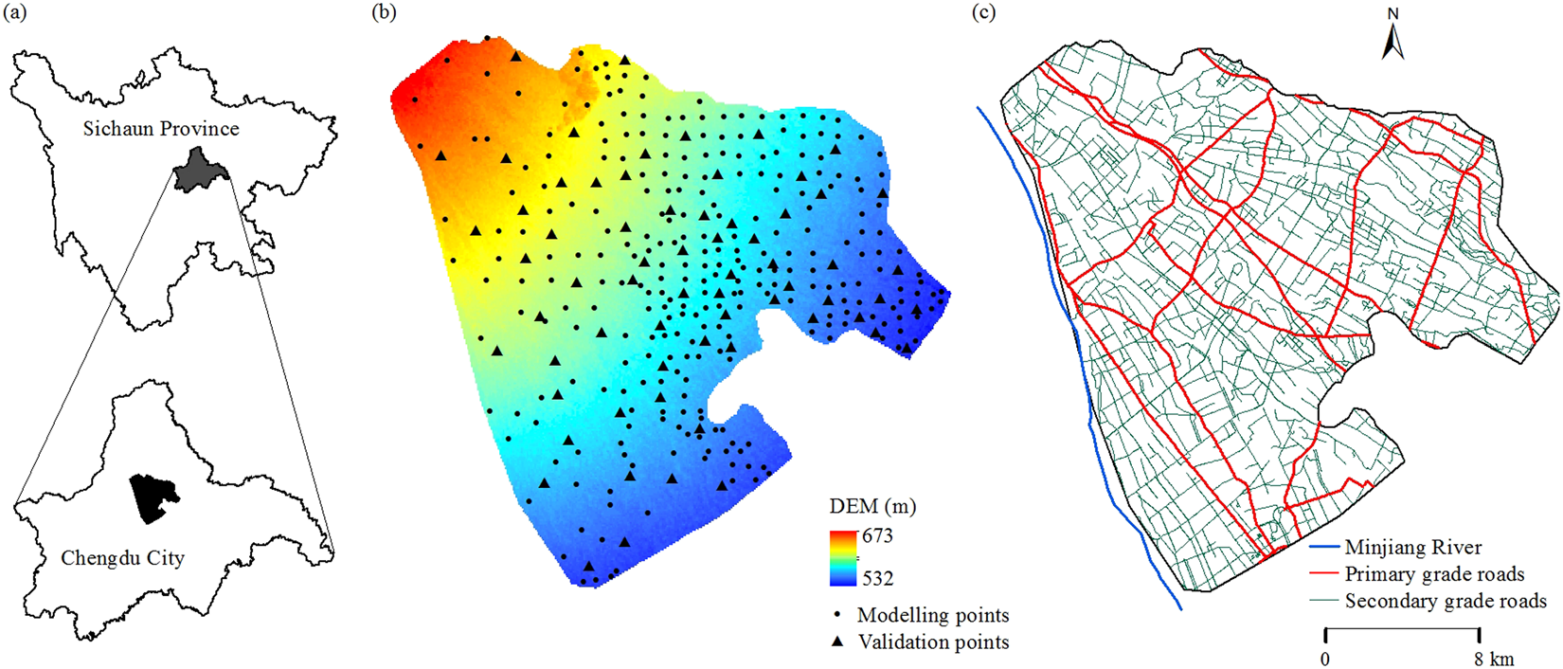
The location of the study area in Sichuan Province (**a**), the digital elevation model (DEM) and the soil sample distribution in the study area (**b**), and the spatial distribution of roads and the Minjiang River (**c**). All the maps were generated in ArcGIS10.1, http://www.esrichina-bj.cn/softwareproduct/ArcGIS/.

High values of soil Cd were found along roads (Fig. 1c and d), which is consistent with other studies^9, 36^. For instance, Zhang reported that Cd is a priority concern as it has the highest contamination factor along the Qinghai–Tibet highway^36^. Khan found that soil Cd in the roadside soils is related to the road grades; the soil Cd is highest in the roadside soils of primary roads, followed by those of secondary and tertiary roads^9^. In the present study, primary roads were found to have a more far-reaching impact on soil Cd (Fig. 1c and d), mainly because of the heavier traffic flows. However, the density of secondary roads was much higher than that of primary roads in the study area (Fig. 3c), which resulted in the fact that the density of secondary roads could explain more of the soil Cd variability than primary roads (Table 1).

### The effectiveness of the environmental covariates for improving the prediction

Soil is the product of complex interactions between environmental factors, such as terrain factors, land use and parent material^6, 12^. The spatial distribution of soil properties may vary significantly within a short horizontal distance due to various environmental factors^13, 16^. It is difficult to obtain accurate predictions in the absence of the environmental auxiliary variables.

Many researchers have shown that the use of the auxiliary information could improve the accuracy of the predictions^5, 12–17^. However, the commonly used factors are not the most effective auxiliary variables for the prediction methods in this plain area due to the gently undulating terrain, homogeneous parent material and soil type in our study area. The results of a correlation analysis suggested that the geological origin might determine the overall spatial trends of the soil Cd distribution, while crop rotation systems and traffic contributed further local variability across the study area (Tables 1 and 4). These obviously influential factors cannot be ignored in an effort to produce a more accurate spatial distribution map of soil Cd. In this study, the environmental covariates were derived from these obviously influential factors and used as auxiliary variables for the prediction methods. The results showed that the methods that employed the derived environmental factors as auxiliary variables (including RK, HASM_RBFNN and HASM_RBFNNs) obtained a higher degree of accuracy and greater detail than the prediction results from OK, which only predicted soil Cd from the neighbouring sampling points (Table 3 and Fig. 2), a finding that was consistent with previous studies^5, 12–17^. This result suggested that the derived environmental variables were effective for improving the prediction results and it was feasible for our approach to derive the auxiliary variables from the obviously influential factors.

### The performance of HASM_RBFNN for reducing predictive error

HASM_RBFNN showed the best performance among the four prediction methods (Table 3 and Fig. 2), which could be attributed to the following factors. First, the RBFNN model has been proven to be more effective than MLR in capturing the relationships between soil and the environmental factors^12, 15^. Other researchers have also found that MLR was not appropriate because the MLR model with inclusion of auxiliary information may deteriorate the spatial structure of the target soil property^37^. In the study area, the relationships between soil Cd and the environmental variables were complex and included a curvilinear relationship between soil Cd and the distance to the Minjiang River (Table 1 and Fig. 1), which suggested that the artificial neural network model was more appropriate. Second, the output of HASM satisfies the iteration stopping criterion, which is determined by the application requirement for accuracy^5^. Typically, soil heavy metal contents of samples from high pollution risk areas are local spatial outliers^38^. This phenomenon was also found in the much higher contents of soil Cd along the roads (Fig. 1c and d) in our study. OK has a smoothing effect and predicts soil Cd content based on the neighbouring soil samples, which underestimates the local high values and overestimates the values around the samples with higher values^1^. In contrast, HASM is a new technique based on a fundamental theorem of surfaces that can generate less error in areas with high local variability through its algorithm and a large enough iteration number^16, 22^, which leads to both HASM_RBFNN and HASM_RBFNN_s_ having larger prediction ranges than those of RK and OK and smaller areas with high values (>0.32 mg·kg^−1^) in the prediction maps (Fig. 2). Finally, the spatial non-stationarity of the relationships between soil Cd and the derived environmental covariates was considered in HASM_RBFNN. The superiority of this consideration could be easily demonstrated from the prediction results along the roads in our study. Although the density of primary roads had significant impacts on soil Cd (Table 2), the cardinal values of soil Cd content were largely different in the two subareas due to the different geological background values (Figs 1a and 3e). This condition resulted in overestimation along primary roads beyond 10 km of the Minjiang River and underestimation along primary roads within 10 km of the Minjiang River when only one model was used to capture the relationships between soil Cd and the environmental covariates across the entire area. This underestimation finally led to an inaccurate estimation of soil Cd along the roads and narrower prediction ranges of HASM_RBFNN_s_ and RK than that of HASM_RBFNN.

### Limitations

Although the selected environmental factors had significant impacts on soil Cd distribution, the relatively low correlation coefficients between soil Cd and the four factors suggested the complexity of the relationships between soil Cd and the environmental factors, and other influential factors, such as fertilization management for specific locations and the distribution of chemical factories that could lead to high local variations of soil Cd on the Chengdu Plain^33^, were not included due to a lack of data. Employing more relevant environmental factors as auxiliary variables could further improve the prediction accuracy. Moreover, the resolution of environmental covariates and the best size of the grid that was used to calculate the road density should be further determined based on the prediction results. These limitations should be considered in the future studies.

## Methods

### HASM_RBFNN and HASM_RBFNN_s_

The observation of soil Cd at the soil sampling point is divided into two components, which can be expressed as

1 $$Z({x}_{i},{y}_{j})=f({x}_{i},{y}_{j})+r({x}_{i},{y}_{j})$$

where *Z*(*x*_*i*_, *y*_*j*_) is the measured soil Cd content at sampling point (*x*_*i*_, *y*_*j*_); (*x*_*i*_, *y*_*j*_) are the coordinates; *f*(*x*_*i*_, *y*_*j*_) is the predicted soil Cd content based on thevarious environmental factors, while *r*(*x*_*i*_, *y*_*j*_) is the residual that is the spatially variable but dependent component; the residual is computed by subtracting *f*(*x*_*i*_, *y*_*j*_) from the original value of soil Cd content. The two components are assumed to be mutually independent and can be predicted by RBFNN and HASM, respectively. The RBFNN model was used to predict *f*(*x*_*i*_, *y*_*j*_) from the environmental covariates as follows:

2 $$f({x}_{i},{y}_{j})=RBFNN({X}_{1},{X}_{2},{X}_{3},{X}_{4})$$

where X_1_, X_2_, X_3_ and X_4_ represent the distance to the Minjiang River, the densities of primary and secondary roads, and NDVI at sampling point (*x*_*i*_, *y*_*j*_), respectively. The RBFNN model includes three different layers^12^: a layer of input neurons providing input variables to the network, a hidden layer of RBF neurons that are directly connected to the output layer, and a layer of output neurons. The Gaussian function is the most commonly used RBF as the activation function of the hidden layer and can be expressed as follows:

3 $${\psi }_{i}(x)=\exp (\frac{-{\Vert x-{u}_{i}\Vert }^{2}}{2{\sigma }_{i}^{2}})$$

where *ψ*_*i*_(*x*) is the radial basis activation function of the hidden layer, *x* is the input vector, *u*_*i*_ is the central vector of the *i*th hidden node, and *σ* is the width of the basis function (spread constant). The number of hidden layer neurons and the width are the two key parameters that must be configured for specific studies. The output layer neuron is a weighted linear combination of RBFs in the hidden layer and can be calculated as follows:

4 $${y}_{j}(x)=\sum _{i=1}^{n}{w}_{ji}{\psi }_{i}(x)+{b}_{j}$$

where *y*_*j*_(*x*) denotes output values of the *j*th node in the output layer, *n* is the number of hidden nodes, *w*_*ji*_ is the connecting weight between the *j*th hidden node and *i*th output node, and *b*_*j*_ is the bias parameter of the *j*th output node.

HASM was used to predict the spatial distribution of *r*(*x*_*i*_, *y*_*j*_), which was developed in terms of a fundamental theorem of surfaces^5, 16, 24–26^ where a surface (*z* = (*x*, *y*, *u*(*x*, *y*))) can be uniquely defined by the first and second fundamental coefficients, which are formulated as follows:

5 $$\{\begin{array}{c}E=1+{u}_{x}^{2}\\ F={u}_{x}{u}_{y}\\ G=1+{u}_{y}^{2}\end{array}{\rm{and}}\{\begin{array}{c}L=\frac{{u}_{xx}}{\sqrt{1+{u}_{x}^{2}+{u}_{y}^{2}}}\\ M=\frac{{u}_{xy}}{\sqrt{1+{u}_{x}^{2}+{u}_{y}^{2}}}\\ N=\frac{{u}_{yy}}{\sqrt{1+{u}_{x}^{2}+{u}_{y}^{2}}}\end{array}$$

The basic theoretical equations of HASM could be formulated as

6 $$\{\begin{array}{c}{u}_{xx}={\Gamma }_{11}^{1}{u}_{x}+{\Gamma }_{11}^{2}{u}_{y}+\frac{L}{\sqrt{EG-{F}^{2}}}\\ {u}_{yy}={\Gamma }_{22}^{1}{u}_{x}+{\Gamma }_{22}^{2}{u}_{y}+\frac{N}{\sqrt{EG-{F}^{2}}}\end{array}$$

where $${\Gamma }_{11}^{1}=\frac{1}{2}(G\cdot E{}_{x}-2F\cdot {F}_{x}+F\cdot {E}_{y})\cdot {(E\cdot G-{F}^{2})}^{-1}$$, $${\Gamma }_{11}^{2}=\frac{1}{2}(2E\cdot F{}_{x}-E\cdot {E}_{y}-F\cdot {E}_{x})\cdot {(E\cdot G-{F}^{2})}^{-1}$$, $${\Gamma }_{22}^{1}=\frac{1}{2}(2G\cdot {F}_{y}-G\cdot {G}_{x}-F\cdot {G}_{y})\cdot {(E\cdot G-{F}^{2})}^{-1}$$, $${\Gamma }_{22}^{2}=\frac{1}{2}(E\cdot G{}_{y}-2F\cdot {F}_{y}+F\cdot {G}_{x})\cdot {(E\cdot G-{F}^{2})}^{-1}$$.

If the maximum lengths of the computational domain in the *x* and *y* directions are respectively *L*_*x*_ and *L*_*y*_, the computational domain can be included in the rectangular domain $[0,{L}_{x}]\times [0,{L}_{y}]$. If *h* represents interpolation step length and I + 2 and J + 2 represent the lattice numbers in direction *x* and in direction *y*, the central point of lattice $(0.5h+(i-1)h,0.5h+(j-1)h)$ could be expressed as $({x}_{i},{y}_{j})$, in which $i=0,1,\cdots ,{\rm{I}},{\rm{I}}+1$ and $j=0,1,\cdots ,{\rm{J}},{\rm{J}}+1$. $u(x+h,y)$ and $u(x-h,y)$ could be formulated as the following Taylor expansio’n in series,

7 $$u(x+h,y)=u(x,y)+h\frac{\partial u(x,y)}{\partial x}+\frac{{h}^{2}}{2!}\frac{{\partial }^{2}u(x,y)}{\partial {x}^{2}}+\frac{{h}^{3}}{3!}\frac{{\partial }^{3}u(x,y)}{\partial {x}^{3}}+{\rm{{\rm O}}}({{h}}^{4})$$

8 $$u(x-h,y)=u(x,y)-h\frac{\partial u(x,y)}{\partial x}+\frac{{h}^{2}}{2!}\frac{{\partial }^{2}u(x,y)}{\partial {x}^{2}}-\frac{{h}^{3}}{3!}\frac{{\partial }^{3}u(x,y)}{\partial {x}^{3}}+{\rm{{\rm O}}}({h}^{4})$$

Formulation (7) minus formulation (8) gives that,

9 $$u(x+h,y)-u(x-h,y)=2h\frac{\partial u(x,y)}{\partial x}+\frac{2{h}^{3}}{3!}\frac{{\partial }^{3}u(x,y)}{\partial {x}^{3}}+{\rm{{\rm O}}}({h}^{5})$$

Therefore,

10 $${u}_{x}(x,y)=\frac{\partial u(x,y)}{\partial x}=\frac{u(x+h,y)-u(x-h,y)}{2h}-\frac{{h}^{2}}{3!}\frac{{\partial }^{3}u(x,y)}{\partial {x}^{3}}+{\rm{{\rm O}}}({h}^{4})$$

For sufficiently small *h*, the finite difference approximation of ${u}_{x}(x,y)$ and ${u}_{y}(x,y)$ could be expressed as,

11 $${u}_{x}(x,y)\approx \frac{u(x+h,y)-u(x-h,y)}{2h}$$

12 $${u}_{y}(x,y)\approx \frac{u(x,y+h)-u(x,y-h)}{2h}$$

Formulation (11) plus formulation (12) gives that,

13 $$u(x+h,y)+u(x-h,y)=2u(x,y)+\frac{2{h}^{2}}{2!}\frac{{\partial }^{2}u(x,y)}{\partial {x}^{2}}+{\rm{{\rm O}}}({h}^{4})$$

Therefore,

14 $${u}_{xx}(x,y)=\frac{{\partial }^{2}u(x,y)}{\partial {x}^{2}}=\frac{u(x+h,y)-2u(x,y)+u(x-h,y)}{{h}^{2}}+{\rm{{\rm O}}}({h}^{2})$$

For sufficiently small *h*, the finite difference approximation of ${u}_{xx}(x,y)$ and ${u}_{yy}(x,y)$ could be expressed as,

15 $${u}_{xx}(x,y)\approx \frac{u(x+h,y)-2u(x,y)+u(x-h,y)}{{h}^{2}}$$

16 $${u}_{yy}(x,y)\approx \frac{u(x,y+h)-2u(x,y)+u(x,y-h)}{{h}^{2}}$$

If $\{{\bar{u}}_{i,j}\}$ are the sampled values of *u* at sampling points $\{({x}_{i},{y}_{j})\}$, ${u}_{i,j}^{n}$ ($n\ge 0$, $0\le i\le {\rm{I}}+1$ and $0\le j\le {\rm{J}}+1$) are the *n*th iteration values of lattices whose centres are points of $({x}_{i},{y}_{j})$, in which ${u}_{i,j}^{0}={\tilde{u}}_{i,j}$ and $\{{\tilde{u}}_{i,j}\}$ are the interpolated values based on the sampled values $\{{\bar{u}}_{i,j}\}$. In terms of numerical mathematics, the (*n* + *1*) th iterative formulation of finite difference of basic equations of HASM given by (6) could be formulated as,

17 $$\frac{{u}_{i+1,j}^{n+1}-2{u}_{i,j}^{n+1}+{u}_{i-1,j}^{n+1}}{{h}^{2}}={({{\rm{\Gamma }}}_{11}^{1})}_{i,j}^{n}\frac{{u}_{i+1,j}^{n}-{u}_{i-1,j}^{n}}{2h}+{({{\rm{\Gamma }}}_{11}^{2})}_{i,j}^{n}\frac{{u}_{i,j+1}^{n}-{u}_{i,j-1}^{n}}{2h}+\frac{{L}_{i,j}^{n}}{\sqrt{{E}_{i,j}^{n}+{G}_{i,j}^{n}-1}}$$

18 $$\frac{{u}_{i,j+1}^{n+1}-2{u}_{i,j}^{n+1}+{u}_{i,j-1}^{n+1}}{{h}^{2}}={({{\rm{\Gamma }}}_{22}^{1})}_{i,j}^{n}\frac{{u}_{i+1,j}^{n}-{u}_{i-1,j}^{n}}{2h}+{({{\rm{\Gamma }}}_{22}^{2})}_{i,j}^{n}\frac{{u}_{i,j+1}^{n}-{u}_{i,j-1}^{n}}{2h}+\frac{{N}_{i,j}^{n}}{\sqrt{{E}_{i,j}^{n}+{G}_{i,j}^{n}-1}}$$

where $$n\ge 0$$, $$0 < i < {\rm{I}}+1$$, $$0 < j < {\rm{J}}+1$$,$${E}_{i,j}^{n}=1+{(\frac{{u}_{i+1,j}^{n}-{u}_{i-1,j}^{n}}{2h})}^{2},$$

$${F}_{i,j}^{n}=(\frac{{u}_{i+1,j}^{n}-{u}_{i-1,j}^{n}}{2h})(\frac{{u}_{i,j+1}^{n}-{u}_{i,j-1}^{n}}{2h}),\,{G}_{i,j}^{n}=1+{(\frac{{u}_{i,j+1}^{n}-{u}_{i,j-1}^{n}}{2h})}^{2},$$

$${L}_{i,j}^{n}=\frac{{u}_{i+1,j}^{n}-2{u}_{i,j}^{n}+{u}_{i-1,j}^{n}}{{h}^{2}\sqrt{1+{(\frac{{u}_{i+1,j}^{n}-{u}_{i-1,j}^{n}}{2h})}^{2}+{(\frac{{u}_{i,j+1}^{n}-{u}_{i,j-1}^{n}}{2h})}^{2}}},$$

$${N}_{i,j}^{n}=\frac{{u}_{i,j+1}^{n}-2{u}_{i,j}^{n}+{u}_{i,j-1}^{n}}{{h}^{2}\sqrt{1+{(\frac{{u}_{i+1,j}^{n}-{u}_{i-1,j}^{n}}{2h})}^{2}+{(\frac{{u}_{i,j+1}^{n}-{u}_{i,j-1}^{n}}{2h})}^{2}}},$$

$${({{\rm{\Gamma }}}_{11}^{1})}_{i,j}^{n}=\frac{{G}_{i,j}^{n}({E}_{i+1,j}^{n}-{E}_{i,j}^{n})-2{F}_{i,j}^{n}({F}_{i+1,j}^{n}-{F}_{i-1,j}^{n})+{F}_{i,j}^{n}({E}_{i,j+1}^{n}-{E}_{i,j-1}^{n})}{4({E}_{i,j}^{n}{G}_{i,j}^{n}-{({F}_{i,j}^{n})}^{2})h},$$

$${({{\rm{\Gamma }}}_{22}^{1})}_{i,j}^{n}=\frac{2{G}_{i,j}^{n}({F}_{i,j+1}^{n}-{F}_{i,j-1}^{n})-{G}_{i,j}^{n}({G}_{i+1,j}^{n}-{G}_{i-1,j}^{n})-{F}_{i,j}^{n}({G}_{i,j+1}^{n}-{G}_{i,j-1}^{n})}{4({E}_{i,j}^{n}{G}_{i,j}^{n}-{({F}_{i,j}^{n})}^{2})h},$$

$${({{\rm{\Gamma }}}_{11}^{2})}_{i,j}^{n}=\frac{2{E}_{i,j}^{n}({F}_{i+1,j}^{n}-{F}_{i-1,j}^{n})-{E}_{i,j}^{n}({E}_{i,j+1}^{n}-{E}_{i,j-1}^{n})-{F}_{i,j}^{n}({E}_{i+1,j}^{n}-{E}_{i-1,j}^{n})}{4({E}_{i,j}^{n}{G}_{i,j}-{({F}_{i,j}^{n})}^{2})h},$$

$${({{\rm{\Gamma }}}_{22}^{2})}_{i,j}^{n}=\frac{{E}_{i,j}^{n}({G}_{i,j+1}^{n}-{G}_{i,j-1}^{n})-2{F}_{i,j}^{n}({F}_{i,j+1}^{n}-{F}_{i,j-1}^{n})+{F}_{i,j}^{n}({G}_{i+1,j}^{n}-{G}_{i-1,j}^{n})}{4({E}_{i,j}^{n}{G}_{i,j}^{n}-{({F}_{i,j}^{n})}^{2})h},$$

${u}_{0,j}^{n+1}={u}_{0,j}^{0}$ ($0\le j\le {\bf{J}}+1$), ${u}_{i,0}^{n+1}={u}_{i,0}^{0}$ ($0\le i\le {\bf{I}}+1$), ${u}_{{\bf{I}}+1,j}^{n+1}={u}_{{\bf{I}}+1,j}^{0}$ ($0 < j < {\bf{J}}+1$), ${u}_{i,{\bf{J}}+1}^{n+1}={u}_{i,{\bf{J}}+1}^{0}$ ($0 < i < {\bf{I}}+1$), ${u}_{0,j}^{n+1}$, ${u}_{i,0}^{n+1}$, ${u}_{{\bf{I}}+1,j}^{n+1}$ and ${u}_{i,{\bf{J}}+1}^{n+1}$ are boundary conditions of HASM.

The matrix formulation of HASM master equations can be respectively expressed as,

19 $${A}_{1}{U}^{n+1}={b}_{1}^{n}$$

20 $${A}_{2}{U}^{n+1}={b}_{2}^{n}$$

where ${U}^{n+1}={({u}_{1,1}^{n+1},\cdots ,{u}_{1,J}^{n+1},{u}_{2,1}^{n+1},\cdots ,{u}_{2,J}^{n+1},\cdots \cdots \cdots ,{u}_{I-1,1}^{n+1},\cdots ,{u}_{I-1,J}^{n+1},{u}_{I,1}^{n+1},\cdots ,{u}_{I,J}^{n+1})}^{T}$, *A*_*1*_ and ${b}_{1}^{n}$ are respectively coefficient matrix and right-hand item vector of Eq. (17), *A*_2_ and ${b}_{2}^{n}$ are respectively coefficient matrix and right-hand item vector of Eq. (18).

If $$Z=[\begin{array}{c}{A}_{1}\\ {A}_{2}\end{array}]$$, $${q}^{n}=[\begin{array}{c}{b}_{1}^{n}\\ {b}_{2}^{n}\end{array}]$$, the following equality-constrained least squares problem can be developed to make the interpolated values equal to or approximate to the sampled values at the sampling points,

21 $$\{\begin{array}{c}\min \,{\Vert Z{U}^{n+1}-{q}^{n}\Vert }_{2}\\ s.t.C{U}^{n+1}=d\end{array}$$

where $C(k,(i-1)\cdot J+j)=1$ and $d(k)={\bar{u}}_{i,j}$ which means that the sampled value is ${\bar{u}}_{i,j}$ at the *k* th sampling point $({x}_{i},{y}_{j})$.

To solve the algorithm (21) which is the least squares problem, a positive weight λ is introduced on the basis of the well known method of weights. The parameter λ is the weight of the sampling points and determines the contribution of the sampling points to the simulated surface. For sufficiently large λ, the algorithm (21) can be transferred into unconstrained least squares approximation,

22 $$\mathop{\min }\limits_{f}{\Vert [\begin{array}{c}Z\\ \lambda C\end{array}]{U}^{n+1}-[\begin{array}{c}{q}^{n}\\ \lambda d\end{array}]\Vert }_{2}\,{\rm{or}}\,[\begin{array}{cc}{Z}^{T} & \lambda {C}^{T}\end{array}][\begin{array}{c}Z\\ \lambda C\end{array}]{U}^{n+1}=[\begin{array}{cc}{Z}^{T} & \lambda {C}^{T}\end{array}][\begin{array}{c}{q}^{n}\\ \lambda d\end{array}]$$

In terms of the Gauss-Codazii equation set, the iteration stopping criterion of HASM is formulated as

$${({\phi }_{1y}-{\varphi }_{2x}-{\phi }_{2}P-{\varphi }_{1}Q)}^{2}+{({\phi }_{2x}-{\varphi }_{1y}-{\phi }_{1}Q-{\varphi }_{2}P)}^{2}+{({Q}_{x}+{P}_{y}+{\phi }_{1}{\phi }_{2}-{\varphi }_{1}{\varphi }_{2})}^{2} < {e}_{t}$$

where ${\phi }_{1}=\frac{L}{\sqrt{E}}$, ${\phi }_{2}=\frac{N}{\sqrt{G}}$, ${\varphi }_{1}=\frac{M}{\sqrt{G}}$, ${\varphi }_{2}=\frac{M}{\sqrt{E}}$, $P=\frac{{\sqrt{E}}_{y}}{\sqrt{G}}$, and ${Q}=\frac{{\sqrt{G}}_{x}}{\sqrt{E}}$; e_*t*_ is the iteration stopping criterion of HASM determined by the application requirement for accuracy.

The application of HASM_RBFNN includes four steps: based on the modelling points, two specific RBFNN models were first trained using a different number of hidden layer nodes and spread constants for the two subareas including within and beyond 10 km of the Minjiang River. The best combinations of the two parameters were tested and determined for the RBFNN configurations, which presented a minimum value of RMSE for the validation points. Second, the two trained RBFNN models were used to predict *f*(*x*_*i*_, *y*_*j*_) for the two subareas with the layers of environmental covariates and to calculate the prediction residuals of RBFNN for the modelling points. Third, HASM was then used to predict the spatial distribution of the prediction residuals of RBFNN. Finally, the RBFNN prediction was summed to the result of HASM as the final prediction of HASM_RBFNN.

To evaluate the performance of the method without considering the spatial non-stationarity of the relationships between soil Cd and the derived environmental covariates, HASM_RBFNN_s_ was established, which only trained one specific RBFNN model for the entire study area.

### OK

OK is the commonly used and classical method in soil science that is based on observations of a target soil variable and of corresponding spatial positions. In this study, the experimental semivariogram was fitted using authorized theoretical models, including linear, Gaussian, spherical and exponential models. The model with the smallest residual sum of squares (RSS) was chosen to provide the key parameters for spatial prediction by the Kriging procedure in the Geostatistical Analyst extension in ArcGIS. The semivariogram parameters of the best model are listed in Table 4. For the number of the closest samples of OK, we chose the best one from 5 to 30 with a 5 step interval.

### RK

RK is a commonly used method that can introduce auxiliary environmental variables using a regression model into the kriging system^14, 19^. The implementation of RK involves three steps: establishing a multiple linear regression between the target variable and auxiliary variables, computing the regression residuals by semivariogram and OK, and summing the regression prediction and the OK prediction of the residuals. The process of RK in this study can be summarized as follows:

$${{\rm{Z}}}_{RK}={{\rm{Z}}}_{R}+{\varepsilon }_{OK}$$

where *Z*_*RK*_ is the predicted values of soil Cd content using RK, *Z*_*R*_ is the predicted values of soil Cd content by a special multiple linear regression that used the four derived environmental covariates as independent variables, and *ε*_*OK*_ is the kriging values of the regression residuals by OK with the semivariogram model parameters computed from the residuals (Table 4).

### Assessment of the performance

The prediction performance of each method was evaluated by the difference between the observations and predictions at validation sites using common indices, including the MAE, RMSE and MRE, which were defined as follows:

$$MAE=\frac{1}{n}\sum _{i=1}^{n}|{Z}_{obs(i)}-{Z}_{pred(i)}|$$

$$RMSE=\sqrt{\frac{1}{n}\sum _{i=1}^{n}{({Z}_{obs(i)}-{Z}_{pred(i)})}^{2}}$$

$$MRE=\frac{1}{n}\sum _{i=1}^{n}\frac{|{Z}_{obs(i)}-{Z}_{pred(i)}|}{{Z}_{obs(i)}}\times 100$$

where *n* is number of validation points (herein n = 66), *Z*_*obs* (*i*)_ is the measured value of the *i*th point (mg/kg), and *Z*_*pred* (*i*)_ is the predicted value of the *i*th point (mg/kg). Generally, lower values of *MRE*, *RMSE* and *MRE* indicate higher prediction accuracies.

### Study Area and Data

#### Study area

The study area (30°41′ 39″–30°57′ 10″ N, 103°39′ 58″–103°58′ 36″E) is located in the central part of the Chengdu Plain in the western region of the Sichuan Basin, China. The entire area is a part of the Minjiang River watershed (Fig. 3a). The study area encompasses an area of 480.3 km^2^, and the elevation ranges from 532 to 673 m, with higher elevations in the northwest and lower elevations in the southeast (Fig. 3b). Q4 grey alluvium is the main parent material (more than 98%), and paddy soil (Fe-leachi-Stagnic Anthrosols) is the only soil type in this area according to the National Soil Census data. Farmland and built-up areas are the two main land use types, which account for 71.1% and 25.8% of the entire area, respectively. Rice-wheat rotation and rice-rapeseed rotation are the two main cropping systems in the farmland. Due to the high soil fertility, farmers may plant other crops after the rice harvest and before planting wheat or rapeseed. This area is characterized by developed transport due to the developed economy and the low relief. Road types include expressways, provincial roads, county roads, town roads and country roads, which have different traffic flow levels.

### Soil samples and analysis

A total of 339 sampling sites were determined on the basis of a 1.5 × 1.5 km^2^ grid from January to March in 2013 and were also away from built-up areas, woodland and water areas. Information regarding each site’s geographic coordinates and road conditions were carefully recorded (Fig. 3b). At each site, a topsoil sample (0–20 cm) was collected with three replicates around the sampling site. Each sample was air dried and passed through a 0.149 mm sieve. The soil Cd content of each sample was determined using graphite oven atomic absorption spectrometry after the soil sample had been digested using a four-acid mixture containing HCl, HNO_3_, HF, and HClO_4_. National standard reference materials, blank value assays and parallel determinations were used to verify the accuracy and precision of the measurements. To evaluate the performance of the prediction methods, 80% of the samples (273 samples) were randomly selected as modelling points using the create subset function of the Geostatistical Analyst in ESRI ArcGIS and the other 20% (66 samples) were used as validation points (Fig. 3b).

### Derivation of environmental covariates

Based on previous studies^11, 32^, the geological origin, road distribution and crop rotation systems were selected as the environmental variables in this study. As the Chengdu Plain is an alluvial plain, distance to the main river can reflect the differences of sedimentation processes of the parent materials and the differences of the development levels of the soils^11^. In this study, the distance to the Minjiang River from each grid of the study area was calculated by buffer analysis in ArcGIS and was used as an auxiliary environmental variable. Road distribution data (.shp format) were obtained from the transportation department of Sichuan Province; these data contain information on the name, grade, date of construction, and other characteristics for each road. According to traffic flow, roads in this area were classified into two grades. The first grade includes expressways, provincial roads and county roads, while the second grade includes town and country roads. Correlation analysis showed that the primary and secondary roads had significant impacts on soil Cd content up to 500 m and 100 m away from the roads, respectively (Fig. 1c and d). The density of primary roads within 500 × 500 m grids and the density of secondary roads within 100 × 100 m grids were calculated based on the road distribution data and were used as two other auxiliary variables. The sixteen-day NDVI from MODIS bands was used to represent the different crop rotation systems. Considering the difference of vegetation cover for the two rotation systems, MODIS NDVIs of February (when there is a large difference in vegetation cover between wheat and rapeseed crops), July (when rice has the largest vegetation cover) and December (probable crop between rice harvest and wheat or rapeseed planting) from 2006 to 2012 were selected to calculate the average NDVI value, which was then used as an auxiliary variable. All datasets of the environmental variables were resampled to a 10-m resolution in consideration of the computation time of the prediction methods.
